# Helmet use determinants and barriers among motorcyclists in world: systematic review 

**Published:** 2019-08

**Authors:** Mohammad Saadati, Homayoun Sadeghi Bazargani, Leili Abedi, Mohammad Shirdel, Ramin Rezapour

**Affiliations:** ^*a*^Road Traffic Injury Research Center, Tabriz University of Medical Sciences, Tabriz, Iran.; ^*b*^Kerman University of Medical Science, Kerman, Iran.; ^*c*^Tabriz University of Medical Sciences, Tabriz, Iran.; ^*d*^Iranian Center of Excellence in Health Management, School of Health Management and Medical Informatics, Tabriz University of Medical Sciences, Tabriz, Iran.

## Abstract

**Background:**

Road traffic injuries were introduced as leading cause of death worldwide and 1.2 million ‎people die due to them every year. Motorcyclists are at an increased risk because of their lack ‎of physical protection such as helmet makes them vulnerable to injury. This study aimed to ‎identify helmet use barriers and determinants.‎

**Methods:**

The information obtained in this review was retrieved from electronic databases such as ‎PubMed, Scopus, Science Direct and Web of knowledge databases using keywords. Moreover, ‎manual search of related journals were done. Also references of the selected articles were ‎reviewed to increase the chance of articles finding. Article screening was done using Endnote ‎X8.2. The inclusion criteria were articles with publishing date after 1995, reporting the barriers ‎or facilitators of helmet using. Conference abstracts and proceedings which had reported the ‎effects of helmet on accidents and with a publishing date before 1995 were excluded. ‎

**Results:**

Determinants of helmet use were identified and categorized in four categories as: Individual ‎and psychological factors (such as awareness and positive attitudes towards the compulsory use ‎of helmet, usefulness, older age, having accident background, experienced driving, and being ‎married), road-related factors (such as riding long distances and in urban roads), socio-‎economic factors (such as higher socio-economic condition and education). Another category of ‎determinants was other factors such as different climatic conditions, various motorcycle brands ‎and certain day of week which had different roles as helmet use determinant or barrier in ‎studies.

**Conclusions:**

Governmental and nongovernmental organization dealing with road traffic prevention should ‎ensure that standard helmets are made available and at affordable price to both motorcyclist and ‎passenger. Continuous education on the importance of helmet use to the community should be ‎addressed through media campaigns to improve the knowledge and attitude. However, ‎compatible helmets must be produced to overarch some barriers.‎

**Keywords:**

Helmet use, Motorcyclists, Determinants, Barriers, World

